# Gap Balancing Technique With Functional Alignment in Total Knee Arthroplasty Using the Cuvis Joint Robotic System: Surgical Technique and Functional Outcome

**DOI:** 10.7759/cureus.78914

**Published:** 2025-02-12

**Authors:** Rajashekhar K T, Adarsh Krishna K Bhat, Naveen Biradar, Aniruddha R Patil, Kartik Mangsuli, Amar Patil

**Affiliations:** 1 Orthopaedic Surgery, Apollo Hospitals, Bangalore, IND; 2 Trauma and Orthopaedics, The University of Edinburgh, Edinburgh, GBR; 3 Orthopaedics, Khaja Bandanawaz Institute of Medical Sciences, Gulbarga, IND

**Keywords:** functional alignment, gap balancing, knee arthroplasty, oxford knee score, robotic knee surgery, total knee

## Abstract

Introduction

The application of robotic technologies in total knee arthroplasty (TKA) has widely grown in the past few years. The preoperative CT (computed tomography) scan planning of the knee along with the quantitative soft tissue information recorded and assessed by the robot can be utilized in achieving functional alignment and aid in gap balancing. Gap tension is an important factor influencing the clinical outcome after TKA. This paper describes our technique for gap balancing and functional alignment using a fully autonomous Cuvis joint robotic system.

Methods

A total of 624 knees underwent primary TKA using Cuvis robotic assistance in the time period between November 2023 to April 2024. A total of 360 patients that included 100 males and 260 females were included in the study. All the surgeries were performed by the same surgeon and the same posterior-stabilized (PS)-design prosthesis was implanted. The medial and lateral gaps were balanced using our technique intraoperatively. The patients were followed up at one, three, and six months duration postoperatively, and their knee functional outcomes were analyzed using the Oxford Knee Score (OKS).

Results

A total of 360 patients with a mean age of 64.36 were part of this study. The study shows significant improvement in knee function post surgery. The average preoperative OKS recorded was 15.82, which improved at the postoperative sixth-month follow-up to a mean value of 42.07. There were no patients with poor results as per OKS scores, and no patients required any revision procedures.

Conclusion

The gap balancing technique with functional knee alignment using the Cuvis joint robotic system improved short-term outcomes, with balanced gaps, controlled alignment, and preserved soft tissue tension. No complications were reported, but further long-term, multicenter studies are needed for definitive conclusions.

## Introduction

Alignment techniques in total knee arthroplasty (TKA) are ever-evolving. The most popular school refers to the mechanical axis of the lower limb for all patients [1,2]. While mechanical alignment offers satisfactory implant survival, it is noted that approximately 10-20% of patients remain unsatisfied [3,4].

Other alignment philosophies have emerged to restore knee kinematics with the aim of improving clinical outcomes and reducing dissatisfaction among patients.

The principle of functional alignment (FA) TKA is to restore pre-arthritic alignment and achieve balanced flexion-extension gaps and equal mediolateral tension by adjusting bone resection with some limit and/or minimum soft tissue release using robotic-assisted technology [5]. Robotic assessment platform utilizing three-dimensional preoperative planning with the use of CT scan to assess the bony anatomy in all three planes to adequately match implant size and position. While there are setbacks in identifying the preoperative ligament and soft tissue laxity accurately, the robotic platform does allow a quantifiable intraoperative assessment of laxity and balance.

The creation of symmetrically balanced flexion and extension gaps is a surgical goal of TKA [6]. Conventionally, gap-balancing techniques rely on ligament release prior to the bone cuts. These ligaments release correct fixed deformities and bring the limb into the correct approximate alignment [7]. FA achieves balanced extension-flexion gaps and soft tissue tension by adjusting bone resections, fine-tuning component positioning, and less soft tissue release with robotic system assistance [8].

There are several robotic systems that are in the application, which elevate the precision of bone resection and are outstanding in evaluating the gap size in real time during the surgery [9-13]. This paper aims to describe the surgical technique of gap balancing with functional knee alignment using the Cuvis joint robotic system and analyze the functional knee outcomes in patients with osteoarthritis of the knee with varus deformity. The Cuvis system is an advanced fully automatic robotic system developed by Curexo Inc. (South Korea) and supported by Meril Life Sciences Pvt. Ltd., India.

## Materials and methods

A total of 409 patients underwent TKA with robotic assistance at Apollo Hospitals, Bannerghatta Road, Bangalore, India, during the time period of the study between November 1, 2023, and April 30, 2024, out of which a total of 360 patients with a mean age of 64.36 years satisfied our inclusion criteria. Out of the 360 patients, 264 (73.33%) patients suffered from bilateral knee osteoarthritis and underwent bilateral TKA. The total number of knees operated were a total of 624, with 318 (51%) on the right side and 306 (49%) on the left knee (Table 1). In the context of the bilateral TKA cases, a simple random sampling (SRS) method was employed to select one knee from each patient for analysis, both preoperatively and postoperatively. All the surgeries were performed by the same team headed by a single experienced joint replacement surgeon using a fully autonomous Cuvis robotic system.

**Table 1:** Patient demographics KL Classification: Kellgren-Lawrence Classification, BMI: body mass index (kilogram/meter^2^).

Demographics	Values
1. Age (years)	
Mean	64.36
Range	52-84
2. Gender	
Male	100(27.8%)
Female	260(72.2%)
3. Side	
Right	318(51%)
Left	306(49%)
4. KL Classification	
III stage	236(38%)
IV stage	388(62%)
5. BMI (kg/m^2^)	
Mean	31.16
Range	22-36

The inclusion criteria included the following: a) Patients with symptomatic knee osteoarthritis with a varus deformity, which necessitates a primary TKA. b) Both the patient and the surgeon agree that TKA is the most suitable treatment option. c)The patient has been assessed by the surgeon and is deemed fit for surgery. d) The patient must be able to provide informed consent and commit to following the postoperative rehabilitation and review program. e) No exclusions based on age, body mass index, or other patient demographics. 

The exclusion criteria included the following: a) Patients with inflammatory arthritis, valgus deformity of the knee, previous knee surgery, or trauma history. b) Intraoperative requirement for a more constrained implant. c) The patient is unable or unwilling to sign the informed consent form specific to this study. d) The patient is unable to attend the follow-up program. e) The patient is immobile or has another neurological condition affecting musculoskeletal function. f) The patient has a bone loss that requires augmentation.

The patients were followed up in the first, third, and sixth months postoperative period, and their radiological and clinical outcomes using the Oxford Knee Scores (OKS) were recorded and analyzed.

Institutional Review Board approval from Apollo Hospitals Institutional Ethics Committee was obtained (approval no. 158/2023), and research was conducted as per standards laid down by the 1968 Declaration of Helsinki. Informed written consent was obtained from all patients in the study and for publishing the clinical data.

Preoperative planning

**Figure 1:**
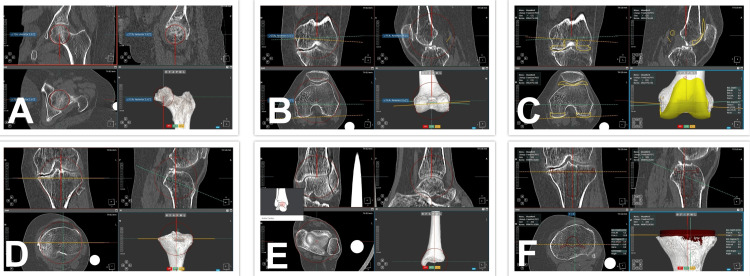
CT images of the hip, knee, and ankle in different planes analysed using the cuvis J Planner software to plan component size and positioning. A: center of the femoral head, B: intercondylar center with the surgical epicondylar axis, C: femoral component planning, D: proximal tibial center, E: centre of the ankle joint, F: tibial component planning

The standing AP, lateral, and skyline X-ray views are taken along with a standing X-ray scanogram of the lower limb. The HKA (hip, knee, and ankle axis), the MPTA (medial proximal tibial angle), the LDFA (lateral distal femoral angle), and the CPAK type were calculated and documented. CT scan with 3D reconstruction of the lower limb was done to visualize the HKA. The scan was further segmented to facilitate the generation of a 3D model using the software of Cuvis Joint J-Planner, and the sites of the femoral and tibial registration were recorded and visualized using the software. Specific bony landmarks were mapped out in multiple planes that include the center of the femoral head, the center of femoral condyles, the transepicondylar axis, the posterior twist angle, and the center of the ankle, which is applied for accurate HKA axis mapping and implant size and position determination (Figure 1). The original placement of the implant was selected based on the principles of mechanical alignment, and afterward, the balance was evaluated and adjusted to achieve functional alignment. All patients with implanted with posterior stabilized (PS) Maxx Freedom® knee system components. The OKS preoperative was calculated and recorded.

Surgical technique

All patients were operated on under spinal anesthesia/general anesthesia + continuous femoral triangle block.

Step 1: Medial Parapatellar Approach to the Knee

**Figure 2:**
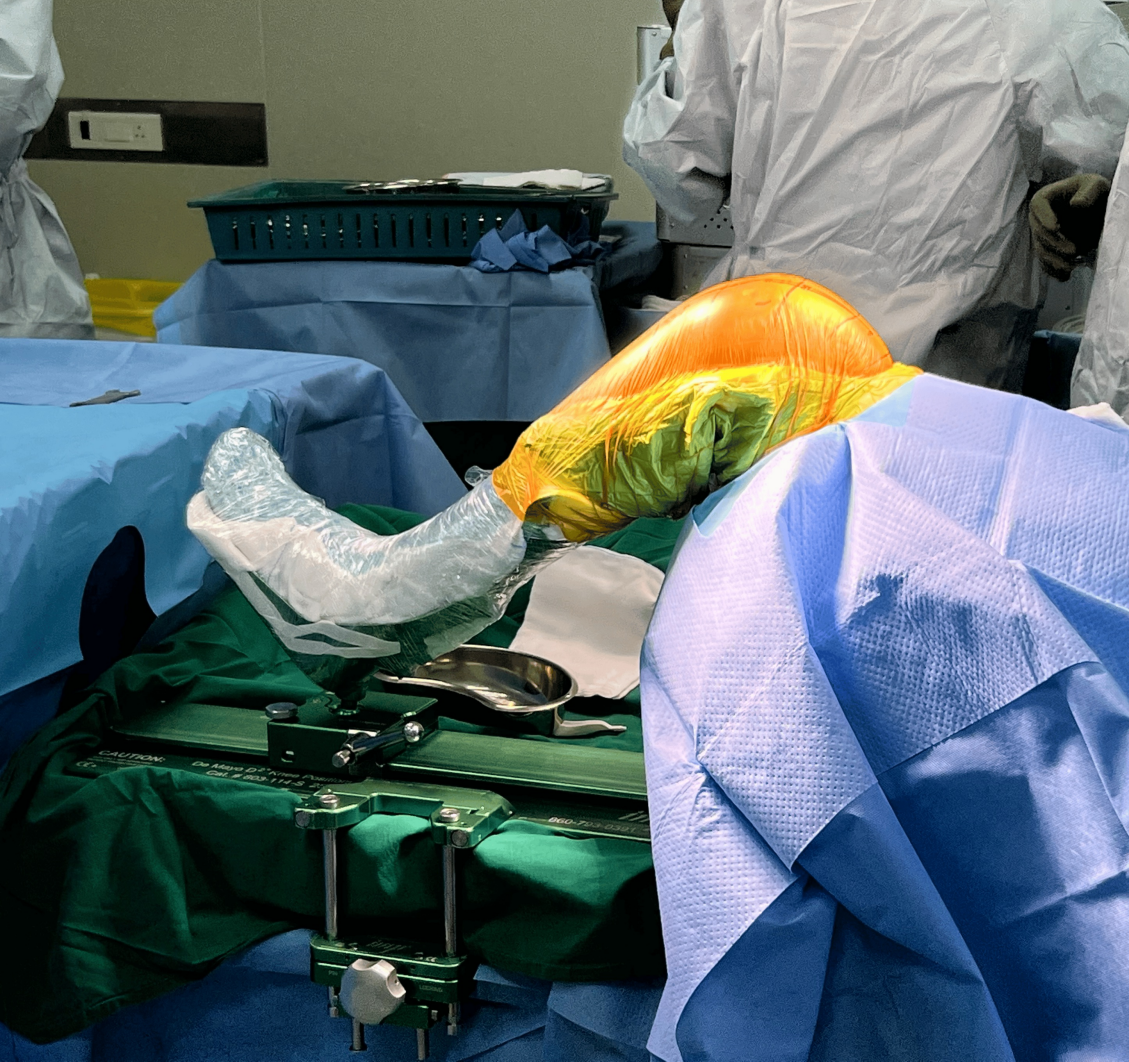
Limb positioned on the De Mayo leg positioner kit.

**Figure 3:**
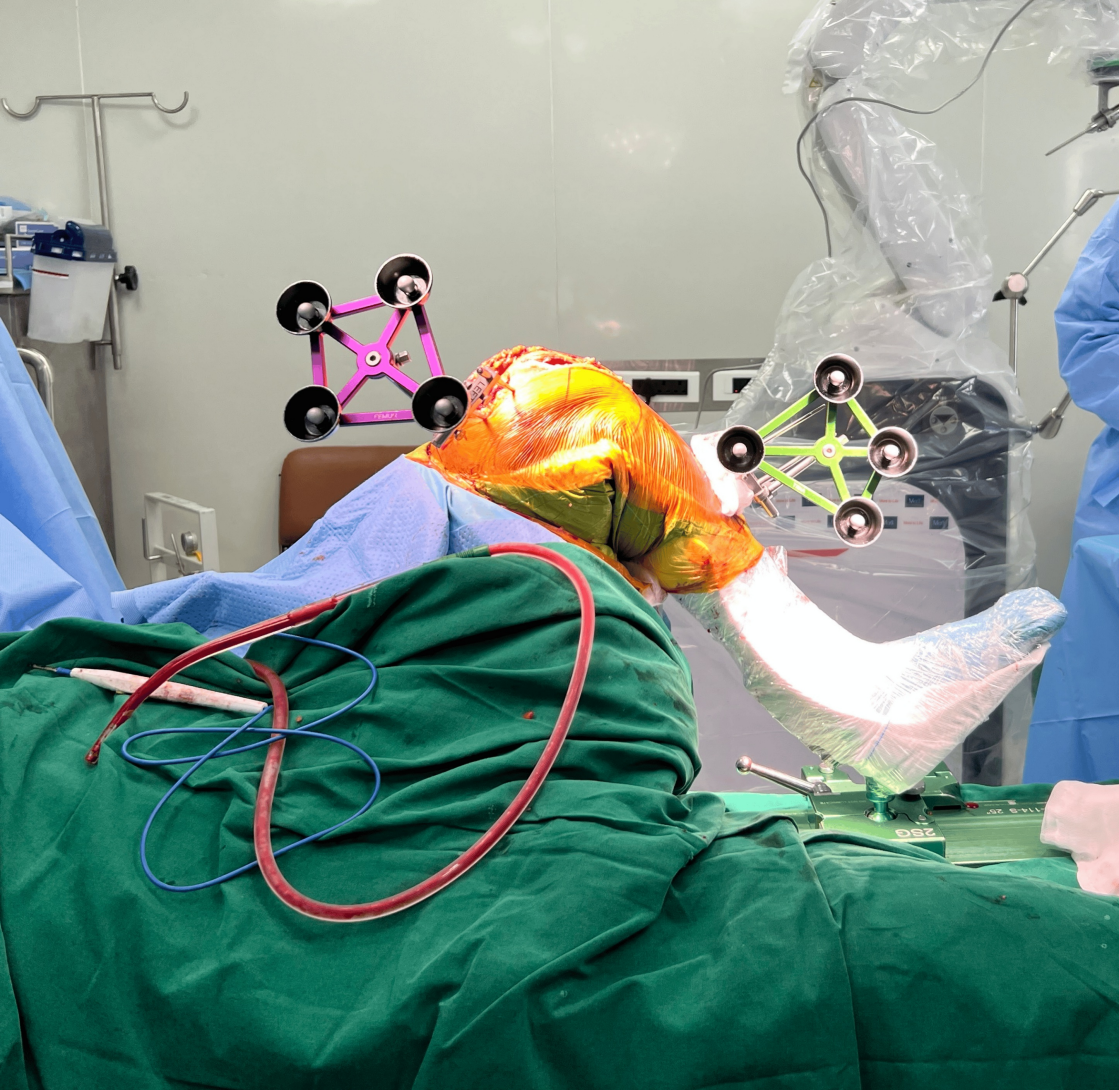
Navigation pins inserted into the distal femur and proximal tibial shaft.

The limb is secured in the De Mayo leg positioner kit (Figure 2). The joint is exposed using the medial parapatellar approach. The navigation pins with reflective arrays are then inserted into the tibial shaft and distal femur as per the company guidelines, and bony landmark registrations of the femoral and tibial surface are done (Figure 3). 

Step 2: Evaluation of the Extension and Flexion Gaps

After the arthrotomy and bony registration, the next step is to nibble the osteophytes that are usually present over the medial aspects of the tibia and femoral surface and release the capsular attachment on the medial aspect of the tibia. This improves the accuracy of gap measurement and ligament tension assessment, as osteophytes may misestimate both gaps and tension.

The native ligament tension and the mediolateral gaps are assessed and recorded intraoperatively with gentle valgus and varus stress in knee extension and 90-degree flexion. The gaps are also assessed in real-time motion from full extension to full flexion of the knee joint to record the dynamic changes in the mediolateral gaps.

*Step 3: Intraoperative Adjustments of Mediolateral Gaps (Extension and Flexion*)

**Figure 4:**
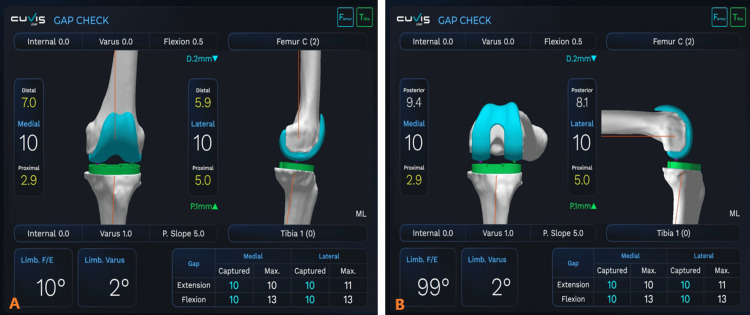
Balanced medial and lateral gaps in knee during extension (10 mm and 10 mm) (A) and knee flexion (10 mm and 10 mm) (B). The Cuvis system interface indicates the preoperative planned adjustments in the femoral component and its size at the top of the figure and preoperative planned adjustments in the tibial component and the size, at the bottom of figure. The intraoperative adjustments are shown at the right corner of the AP image. In this case, we have shifted distal femoral component down by 2 mm and tibial component up by 1 mm, which has resulted in balanced mediolateral gaps. The table at the right-bottom corner of the image shows the maximum gaps obtained after varus and valgus stress test in extension and flexion.

This step aims to create a maximum gap of 10 mm (±1) while giving valgus and varus stress in the medial and lateral gaps, respectively, both in extension (0-15 degrees) and flexion (80-100 degrees) (Figure 4). The values that are displayed by the robot are in fact the distance between the tibial surface and femoral surface with the components and are accounted with the thickness of the prosthesis, effectively displaying the size of the polyethylene insert. The overall alignment (HKA) is maintained within the boundary of a maximum of 5 degrees of limb varus. The joint line obliquity is maintained.

The intraoperative adjustments that are executed based on the different scenarios in a varus knee are listed in Table 2.

**Table 2:** Intraoperative adjustments to achieve mediolateral gap balance.

Intraoperative scenario	Adjustments
Excess gap in both flexion and extension.	Proximalize the tibial component.
Decreased gap in both flexion and extension	Distalize the tibial component.
Balanced extension, tight flexion	1) Downsize the femoral component (anterior referencing). Posterior condylar offset ratio (Posterior condylar offset/femoral diameter) is maintained <95%. 2) Shift the femoral component anteriorly but limit to 2mm to avoid overstuffing of patellofemoral joint. The anterior femoral offset in maintained <15%. 3) Adjust the posterior of slope of the tibia maximum up to 7 degrees. 4) Shift the tibial component distally resulting an increase in both flexion and extension gaps, hence to be followed by distalizing the femoral component to balance extension gap.
Balanced extension, lax flexion	1)Posteriorize (max 1mm) and flex (max 2 degrees) the femoral component to decrease the flexion gap. Avoid anterior notching with careful visualization clinically and using robot monitor. 2) Proximalisation of the tibial component which decreases both extensiom-flexion gaps hence to be followed by proximalisation of the femoral component to increase the extension gaps.
Balanced flexion, lax extension	Distalize the femoral component.
Balanced flexion, tight extension	Proximalize the femoral component.
Tight medial gaps in flexion and extension	Consideration: Check for posteromedial osteophytes in the femur and tibia on the x-rays and if present, anticipate laxity after osteophyte removal. 1) Place the tibial component in varus (max 4 degrees). 2) Perform soft tissue releases in the following order: Deep MCL(Medial collateral ligament), Posterior oblique ligament, Medial reduction osteotomy, Semimembranosus release, Pie crusting of the superficial MCL
Balanced extension, tight medial flexion gap	Consideration: Check for posterior osteophytes on the medial femoral condyles. If present, leave the medial space untouched anticipating laxity after osteophyte removal. If Absent: 1) Externally rotate the femoral component (max 3 degrees to the surgical TEA). 2) Perform soft tissue release medially and pie crusting of the anterior MCL fibers. It is important to note that osteophyte removal can increase both flexion and extension gaps.
Balanced flexion, tight medial extension gap	Place the femoral component in varus (max 3 degrees) and further perform soft tissue release if needed. Based on the gaps, femoral component Is placed within 3 degrees of valgus to 3 degrees of varus.
Tibial posterior slope	Based on the preoperative CT measurements, adjust the posterior slope up to a maximum of 7 degrees to increase the flexion gap.

To summarize, the femoral component is usually kept at 2 degrees maximum valgus to a maximum of 3 degrees of varus and 0 degrees to 3 degrees of external rotation. The tibial component is kept at 0 degrees to 4 degrees of maximum varus. To balance the flexion gap the femoral component is shifted anteroposteriorly depending on the circumstances, a maximum of 2 mm combined with flexion to avoid notching. Femoral component sagittal alignment is maintained so that anterior femoral offset is <15% and posterior condylar offset ratio <95% postoperatively. Values above this are considered as Femoral component oversizing and may be a reason for anterior knee pain [14]. It is important to visualize the chances of anterior notching which is displayed on the screen when adjusting the femoral component posteriorly. The overall HKA is maintained in the range of 0 degrees to 5 degrees of varus. The joint line is maintained within 3 mm of the native joint line.

Step 4: Final Gap Assessment and Resection by the Cuvis Automatic Robot

Once the final gaps are assessed and ascertained after the intraoperative bony adjustments and soft tissue release, the robotic resection is carried forward. Once completed, trial components are inserted and the gaps are reassessed clinically using robotic assistance, followed by cementation and final component implantation. In cases with severe lateral laxity, a gap discrepancy of 2 mm is accepted to avoid erratic component placement or excessive soft tissue release. The joint line obliquity and CPAK type are maintained as in the preoperative stage. The patellar osteophytes are nibbled and tracking is ascertained before closure.

Postoperative protocol

Pain Management

This includes continuous femoral triangle block until postoperative day 2, supplemented with intravenous NSAIDs (non-steroidal anti-inflammatory drugs)/paracetamol/opioids based on the pain intensity. On the postoperative day 2, the femoral block is disconnected, and IV analgesics are switched to oral.

The patient is encouraged to walk with a rigid knee immobilizer brace and walker support for the first few postoperative days, along with knee range of motion exercises, quadriceps, and hamstring strengthening exercises, which are continued for a month duration.

Wound care involves a change in the dressing on postoperative day 3, which is usually the day of discharge. The sutures are removed two weeks from the date of surgery.

Lower limb alignment X-rays and knee X-rays are assessed during the first-month follow-up. The patient is further instructed to come for follow-up after three months, six months, and one year of surgery. The OKS are documented at the time of each visit.

Outcome measurement and statistical analysis

The patients were assessed utilizing the OKS, a patient-reported outcome measure-based questionnaire [15]. As per the latest modifications made available by the Oxford University Innovation, a subsidiary of the University of Oxford, a score of 0 denotes the worst outcome and a maximum of 48 represents the best outcome [16]. Furthermore, the patients are classified into those with excellent outcomes (>41), good (34-41), fair (27-33), and poor (<27).

The data were collected and compiled in MS Excel (Microsoft Corp., Redmond, WA, USA). Descriptive statistics was used to present the data. To analyze the data, IBM SPSS Statistics for Windows, Version 25.0 (IBM Corp., Armonk, NY) was used. The Kolmogorov-Smirnov (KS) test was employed to assess the normality of the data distribution. In the context of the bilateral TKA cases, the SRS method was employed to select one knee from each patient for analysis, both preoperatively and postoperatively. The significance level was fixed as 5% (α = 0.05). Qualitative variables were expressed as frequency and percentages, and quantitative variables were expressed as mean and standard deviation. To compare the pre- and postoperative scores, a paired t-test was used. The rationale for using a paired t-test stems from its ability to compare the means of two related groups or measurements (preoperative and postoperative values).

## Results

A total of 360 patients with an average age of 64.36 years and an average BMI (body mass index) of 31.16 kg/m^2 ^were included in this prospective observational study. The patients were followed up during the first, third, and sixth months postoperative period. The OKS were recorded to document the functional improvement, and standing scanograms were taken to analyze the radiological parameters.

**Figure 5:**
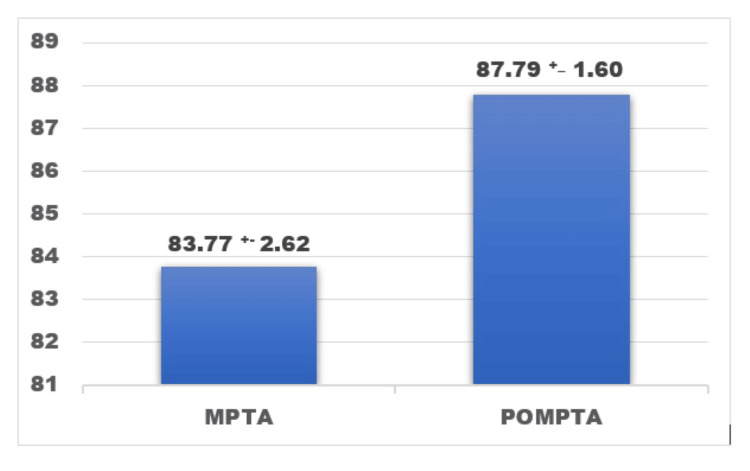
Comparison between the mean preoperative MPTA +/- SD (MPTA) and mean postoperative MPTA +/- SD (POMPTA) in degrees. p-value < 0.001, paired t-test MPTA: mean medial proximal tibial angle, POMPTA: mean postoperative medial proximal tibial angle The figure was obtained from the SPSS software (version 25) and was further edited in Microsoft Word (package version 2021) for the setting of designs and fonts.

**Figure 6:**
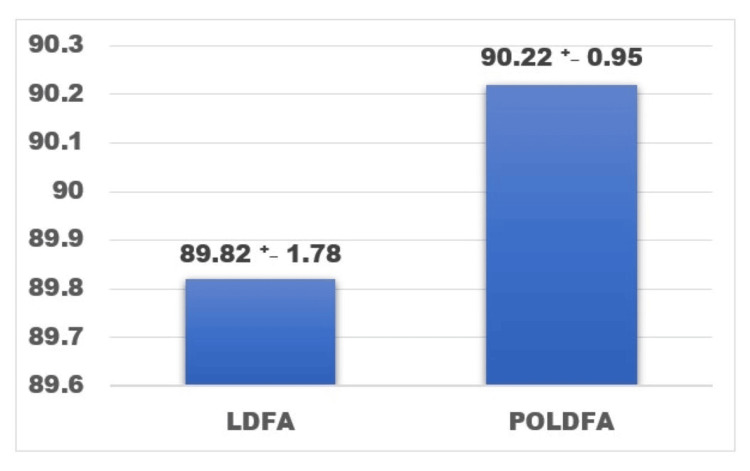
Comparison between the mean preoperative LDFA (LDFA) +/- SD and mean postoperative mean LDFA (POLDFA) +/-SD in degrees. p-value < 0.002, paired t-test LDFA: mean lateral distal femoral angle, POLDFA: mean postoperative lateral distal femoral angle The figure was obtained from the SPSS software (version 25) and was further edited in Microsoft Word (package version 2021) for the setting of designs and fonts.

**Figure 7:**
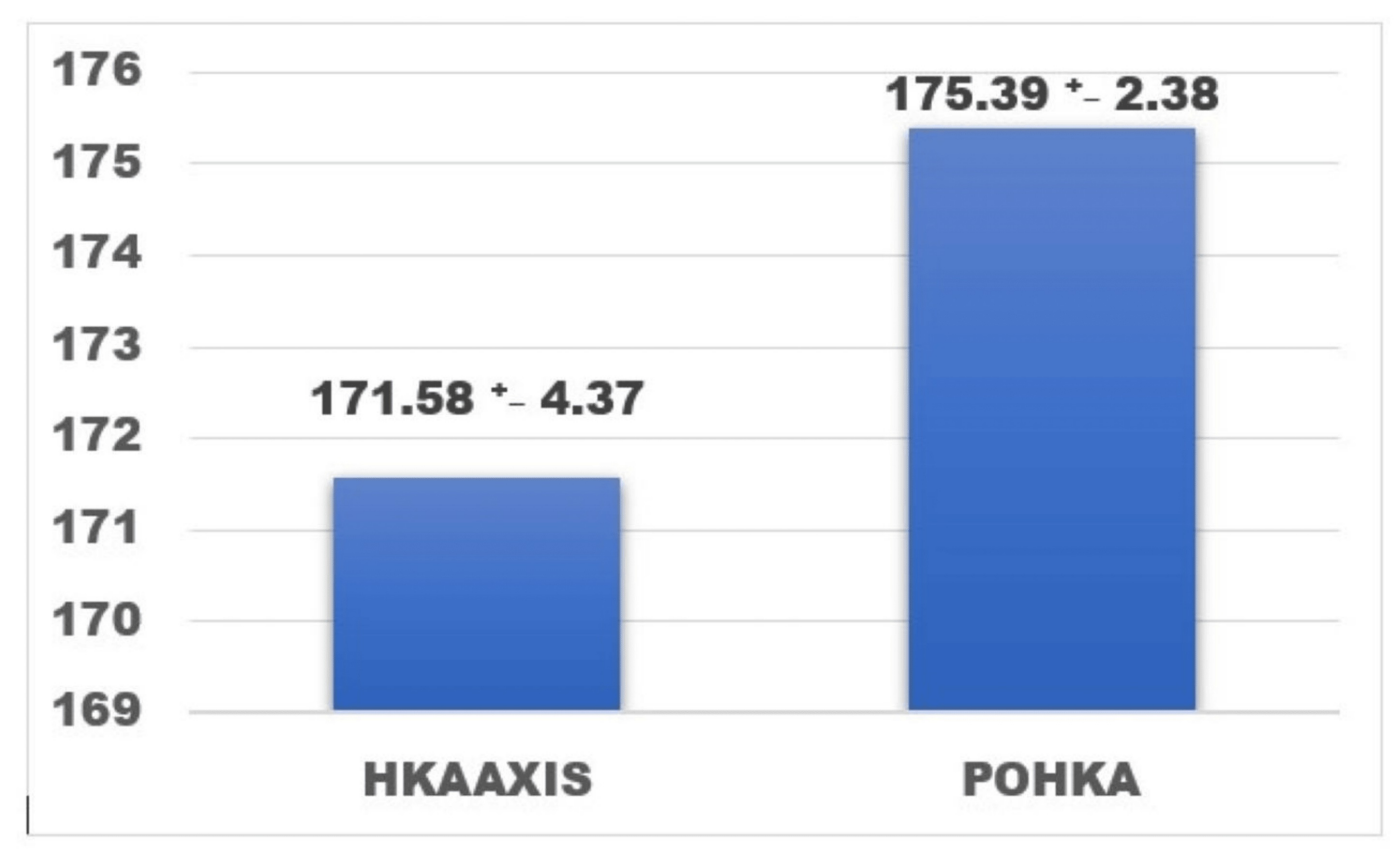
Comparison between the mean preoperative and mean postoperative HKA with SD in degrees. p value < 0.001, paired t-test HKA: mean hip knee ankle axis, POHKA: mean post operative HKA, SD: standard deviation

The preoperative recorded MPTA, LDFA, and HKA were compared to the postoperative values and analyzed. The preoperative mean (SD) MPTA recorded was 83.75 (2.62) compared to the postoperative MPTA (PO-MPTA) recorded value of 87.79 (1.61) with a p-value <0.001 (Figure 5). The LDFA mean values remained the near same preoperatively and postoperatively (PO-LDFA) with values of 89.89 (1.78) degrees and 90.22 (0.95) degrees (Figure 6). The postoperative mean (SD) HKA recorded was 175.39 (2.38) degrees compared to the preoperative mean of 171.58 (4.37), which implies the shift more toward the neutral axis from the preoperative varus (Figure 7).

**Figure 8:**
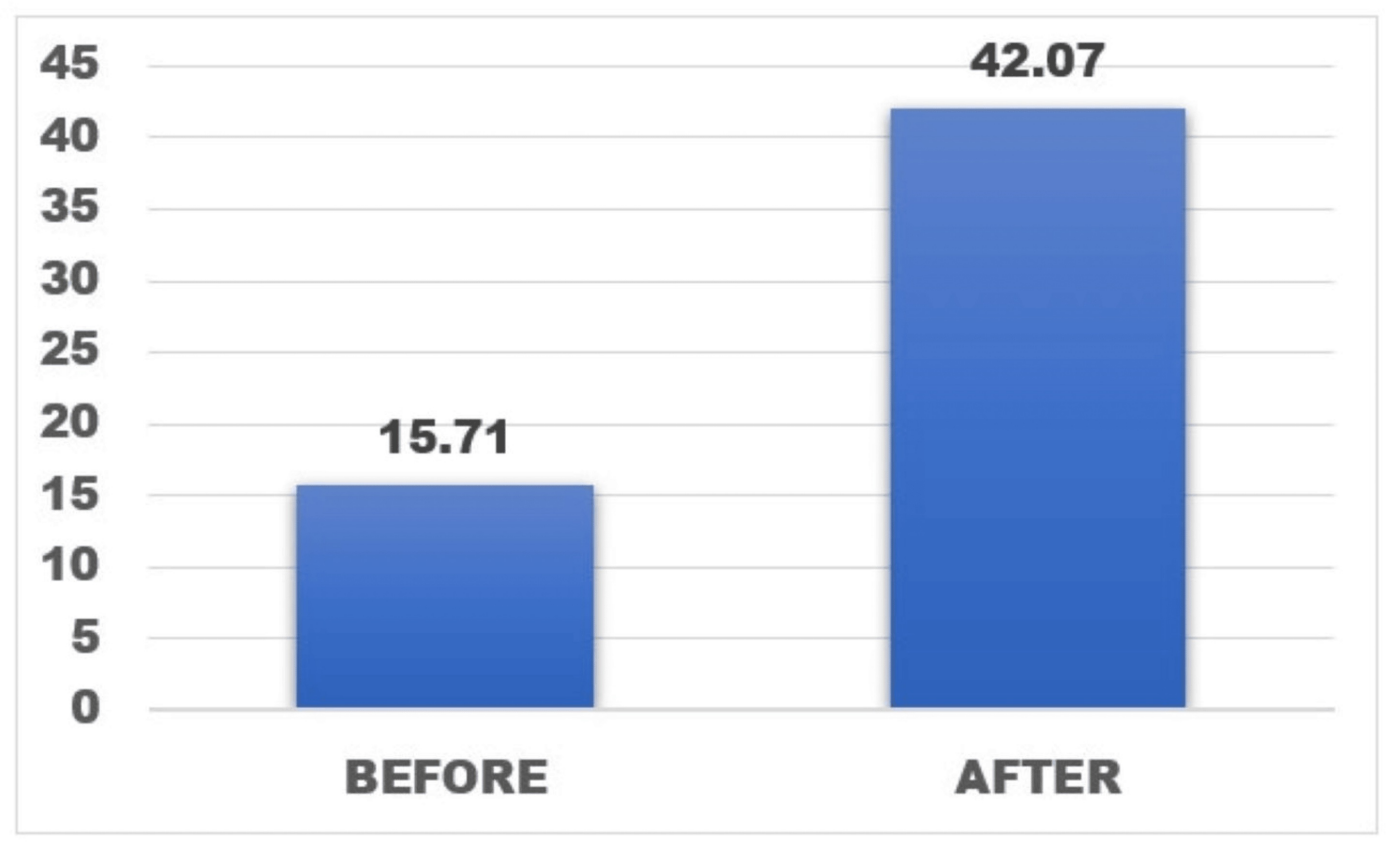
Mean OKS recorded preoperatively and during the sixth-month follow up. p-value < 0.001, paired t-test OKS: Oxford Knee Score The figure was obtained from the SPSS software (version 25) and was further edited in Microsoft Word (package version 2021) for the setting of designs and fonts.

**Figure 9:**
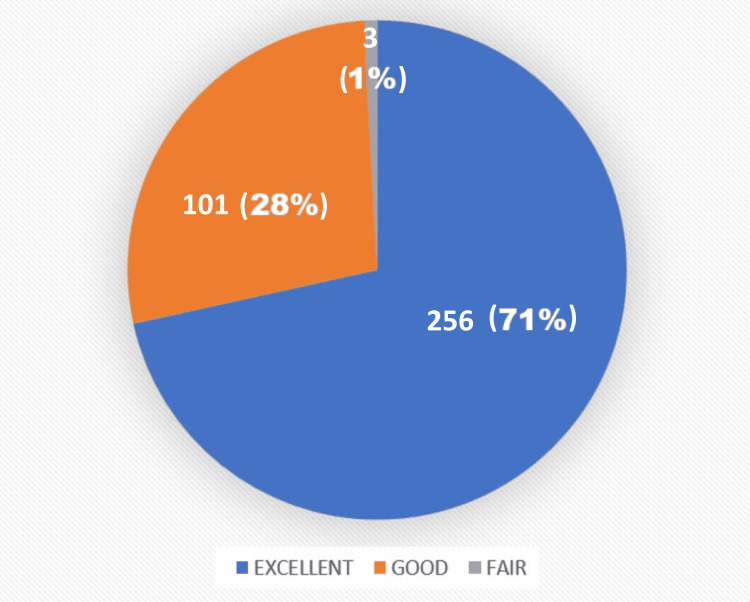
Distribution of the sixth-month postoperative recorded Oxford Knee Scores. OKS (Oxford Knee Scores): excellent (>41), good (34-41), fair (27-33), poor (<27) The figure was obtained from the SPSS software (version 25) and was further edited in Microsoft Word (package version 2021) for the setting of designs and fonts.

The mean preoperative OKS recorded was 15.82, which improved at the postoperative sixth-month follow-up to a mean value of 42.07 (Figure 8). Based on the OKS systems, 71% of the patients recorded excellent results at the sixth-month follow-up. No patient presented with a poor outcome in our study (Figure 9). No patient presented with any untoward complication intraoperatively and during the postoperative follow-up period such as infections, periprosthetic fractures, or need for revision surgeries. No patient was lost to follow-up.

## Discussion

The important observation of the study is that functionally aligned knee with the described gap balancing technique using the Cuvis robotic system is safe and efficient and achieves good to excellent results over a short follow-up period in Indian patients with grade III-IV osteoarthritis.

Currently, in this study, FA safe zones are set from 5° varus to neutral for final HKA, 4° varus to neutral in tibial coronal alignment, 3° of varus to 3° of valgus for femoral component, and rotation of 0° to 3° of femur rotation to the surgical transepicondylar axis (TEA) in a varus osteoarthritic knee. Oussedik et al. in their study addressing different alignment philosophies in TKA suggested placing the tibial component in up to 3° of varus and overall limb alignment to be kept within the 0° ± 3° safe zone of coronal alignment in functional alignment. The study hinted at the possibility of a change in the zones in the future with further research [17]. Clark et al. in their study on functional alignment TKA in constitutionally varus knee suggested coronal alignment be limited to 6° varus to 3° valgus for the tibia component, 6° valgus to 3° varus for the femoral component, and 3° varus to 3° valgus for HKA. With these set boundaries, their study concluded improved outcomes at two years post-TKA [18]. Shatrov et al. suggested FA safe zones to be set from 6° varus to 0° valgus for the final HKA and tibial coronal alignment and 6° valgus to 3° varus for femoral coronal alignment and ±6° to the surgical TEA for femoral rotation for varus knees, which is also the current approved limits set by the US Food and Administration (FDA) [19]. Bellemans et al. demonstrated that 90% of native knees fall within this range [20]. Even though the current US FDA limits for femoral rotation are set at 6° freedom from TEA, increased internal rotation carries a risk of patellar maltracking and anterior femoral notching, which can be catastrophic. Berger et al. in their study concluded that internal component rotation may be the predominant cause of patellofemoral complications in patients with normal axial alignment [21]. 

In patients with proximal tibia varus or bowing of the tibial shaft, the degree of varus correction in the tibial component is limited to 2°. This restriction is implemented to prevent the appearance of limb bowing and to reduce the risk of proximal tibial stress fractures postoperatively. Correction of the preoperative varus deformity is also a factor considered by Indian patients [22], and the reoccurrence of the deformity postoperatively is not well received by all, in our experience.

Patil et al. in their study highlighted the efficacy and precision of TKA using a Cuvis automatic robot. Their findings solidified the safety of robotic-assisted TKA. The disparity in alignment and accuracy between anticipated, planned outcomes, and postoperative outcomes while utilizing Cuvis was found to be equal to or lower than 1 mm or 1° [23]. The findings were similar to the findings in our study; hence, the planned mediolateral gap is 10 mm (±1). Based on the postoperative gaps, keeping in mind the chance of a 1 mm error, a polyethylene insert of one among 9-11 is finally inserted to attain balanced mediolateral gaps and stability, confirmed clinically and using robotic assistance.

Chandrashekar et al. in their case series comprising 500 cases established the intraoperative safety of the Cuvis robotic systems in Indian patients [24].

The findings in this study correspond to the findings present in previous studies in this regard, which have shown successful attainment of high precision and accurate alignment while preserving soft tissues, resulting in favorable functional and clinical outcomes using robotic-assisted TKA [25-27].

Flexion-extension gap balance, soft tissue tension, and overall limb alignment play a very crucial role in influencing patient satisfaction, functional outcome, and long-term survivorship post-TKA [28,29].

Even though there are favorable reports on implant survivorship, multiple studies have demonstrated higher dissatisfaction rates with mechanical alignment [4]. Conventional mechanical alignment techniques aim to achieve neutral alignment; however, for patients with severe varus/valgus deformity, extensive soft tissue may be required to obtain balanced mediolateral gaps, which is considered an important factor that results in postoperative pain and hence dissatisfaction [30,31]. As a result, some researchers have proposed a paradigm shift in the way ideal alignment is to be defined.

Kinematic alignment has recently picked up pace as it can negate the need for extensive soft tissue release. In this technique, the bony cuts and implant positioning are done in order to restore the patient’s pre-arthritic knee anatomy [32,33]. Although there is research supporting better functional outcomes and component survivorship, there is widespread concern regarding implant loosening when positioned erratically in patients with severe deformities. Moreover, there are studies that suggest that kinematic alignment followed by implantation using current TKA implants does not restore trochlear anatomy, which may result in patellofemoral joint problems, warranting the need for specifically designed implants for kinematic TKA [34,35].

Deckey G et al. in their study consisting of 220 cases concluded that robotic-assisted TKA is significantly more accurate and precise in planning both component positioning and final polyethylene insert thickness. However, they pressed on the need for further research on whether this had an impact on the clinical outcomes [9].

Jong et al. in a comparative study of outcomes with FA using robotic assistance concluded that there were superior one-year postoperative outcomes compared to conventional and robotic-assisted mechanically aligned TKA [36].

The following are the limitations of the study: 1) This study has a short duration of follow-up of six months. Long-term further follow-up and analysis are required to strengthen and establish the outcomes using our gap-balancing technique with functional alignment using robotic assistance. 2) The study is a prospective observational study and consists of patients who underwent robotic-assisted functional alignment TKA, and there was no comparison study done between functional alignment and mechanical alignment or any other alignment techniques. The absence of such a comparison limits the ability to draw broader conclusions about which approach-functional alignment, mechanical alignment, or others yields the best outcomes in terms of pain reduction, knee function, longevity of the implant, or overall patient satisfaction. 3) It is a single-center study and the surgeries are performed by a single surgeon; hence, there is a potential for biases related to the surgeon’s skill, patient selection, and center-specific factors. These biases may limit the ability to generalize the findings to other healthcare settings, surgeons, or patient populations. 4) This is a single-arm study and has no control group. As there is no control group, the ability to assess whether the robotic-assisted functional alignment provides superior results compared to other standard or alternative methods is limited. 5) Our sample size is 624 knees, which is comparatively small. Larger multicentric randomized control trials with this technique and also comparative studies with other techniques are required to establish and solidify the findings of this study.

## Conclusions

This gap-balancing technique with the functional alignment of the knee using the fully automatic Cuvis robotic system aids in achieving balanced flexion-extension mediolateral gaps, controlled limb alignment, and preservation of the soft tissue tension, thus contributing to the enhanced patient satisfaction and functional outcomes in the short-term follow-up after TKA. There were no untoward intra- or postoperative complications with this technique. Further long-term and multicentric comparative studies comparing different techniques and alignment principles are required to draw solid conclusions.
